# A review on the research progress of traditional Chinese medicine with anti-cancer effect targeting ferroptosis

**DOI:** 10.1186/s13020-023-00838-1

**Published:** 2023-10-13

**Authors:** Longyan Wang, Huiming Huang, Xingxing Li, Lishan Ouyang, Xuejiao Wei, Jinxin Xie, Dongxiao Liu, Peng Tan, Zhongdong Hu

**Affiliations:** 1 Modern Research Center for Traditional Chinese Medicine, Beijing Research Institute of Chinese Medicine, Beijing University of Chinese Medicine, No. 11 North 3Rd Ring East Road, Chaoyang District, Beijing, 100029 People’s Republic of China; 2 Dongfang Hospital, Beijing University of Chinese Medicine, Beijing, 100078 China

**Keywords:** Traditional Chinese medicine, Anti-tumor, Ferroptosis, Mechanisms

## Abstract

Ferroptosis is a non-apoptotic form of regulated cell death characterized by iron-dependent lipid peroxidation. It can be triggered by various mechanisms, including the glutathione peroxidase 4 (GPX4)-glutathione (GSH) axis, iron metabolism, lipid metabolism, the GTP cyclohydrolase 1 (GCH1)-tetrahydrobiopterin (BH4) pathway, and the ferroptosis suppressor protein 1 (FSP1)-coenzyme Q10 axis. The redox balance is disrupted when ferroptosis occurs in cells, which is fatal to cancer cells. Additionally, some tumor-associated genes are involved in ferroptosis. Hence, targeting ferroptosis might be an effective strategy for treating cancer. Several small-molecule compounds exhibit anti-tumor effects through ferroptosis, including sorafenib and altretamine, which induce ferroptosis by inhibiting System-Xc and GPX4 respectively, but many problems, such as poor druggability, still exist. Some studies have shown that many traditional Chinese medicine (TCM) induce ferroptosis by inhibiting GPX4, solute carrier family 7 member 11 (SLC7A11), and nuclear factor (erythroid-derived 2)-like 2 (Nrf2), or by increasing the expression of Acyl-CoA synthetase long-chain family member 4 (ACSL4), transferrin (TF), and transferrin receptor 1 (TFR1). These changes can lead to the lysosomal degradation of ferritin, accumulation of iron, lipid peroxidation and the production of reactive oxygen species (ROS), which in turn can promote anti-tumor activities or synergistic effects with chemotherapeutic drugs. In this study, we elucidated the underlying mechanisms of ferroptosis, and the anti-tumor pharmacology of TCM targeting ferroptosis including prescriptions, Chinese herbs, extracts, and natural compounds. Our findings might act as valuable reference for research on anti-tumor drugs targeting ferroptosis, especially those drugs developed from TCM.

## Introduction

In 2012, the Whitehead Institute for Biomedical Research, Massachusetts Institute of Technology defined ferroptosis as cell death induced by the increase in iron-dependent lipid peroxidation [1]. When the Nomenclature Committee on Cell Death updated the cell death system, they reclassified ferroptosis as non-apoptotic programmed cell death in 2018 [2]. Ferroptosis is different from other modes of cell death. It is not affected by the suppression of receptor-interacting protein 1/3 and does not require no caspase activation [3]. Ferroptosis is accompanied by the reduction and atrophy of mitochondria, iron accumulation, and lipid peroxidation [4, 5]. These changes are associated with several factors, such as ferritin abundance, glutathione peroxidase 4 (GPX4), and glutathione (GSH)) levels [4].

Cancer is one of the deadliest diseases in the world, affecting more than 10 million people each year [6]. Drug-induced apoptosis of cancer cells is one of the main methods to treat cancer [7]. However, since cancer cells are intrinsically resistant to apoptosis, the effectiveness of cancer treatment by inducing apoptosis is limited [8]. Therefore, as a non-apoptotic cell death process, ferroptosis provides a novel and promising strategy for cancer treatment [8].

Traditional Chinese medicine (TCM) is an effective cancer treatment strategy, that targets ferroptosis through different pathways [9]. For example, curcumin can target the long non-coding RNA axis to promote ferroptosis and exert an anti-lung cancer effect, whereas, Fuzhengkang’ai decoction was found to sensitize cancer cells to ferroptosis by modulating lipid peroxidation and intracellular levels of ferrous ions [10, 11]. In this review, we summarized the mechanisms of ferroptosis in cancer and TCM prescriptions, Chinese herbs, extraction parts, monomers, and derivatives that target ferroptosis against cancer. This study might act as a reference for the research and development of anti-tumor drugs, especially those derived from TCM.

## Mechanisms of ferroptosis

Ferroptosis is characterized by intracellular iron accumulation and polyunsaturated fatty acid peroxidation [7, 12]. Mitochondrial condensation or swelling, and the loss of cristae and mitochondrial membrane potential are the morphological and physiological differences between ferroptosis and other forms of programmed cell death [13]. Reactive oxygen species (ROS) produced by iron-mediated Fenton reaction and Fenton-like reactions cause excessive oxidation of polyunsaturated fatty acids (PUFAs), which lead to lipid peroxidation and free radical chain reaction [14]. Lipoxygenases (LOXs) also catalyze the deoxygenation of PUFAs to produce lipid hydroperoxide, which damages the polyunsaturated phospholipids in the cell membrane, changes membrane fluidity, and increases the permeability of the membrane [7].

The enzyme GPX4 decreases lipid peroxides using GSH as a cofactor. After GSH is depleted or GPX4 becomes inactivated, cells initiate an abnormal process that leads to ferroptosis [15]. Additionally, the abnormal uptake or excretion of iron-related proteins in cells trigger iron accumulation, and high levels of ferrous ions (Fe^2+^) generate large quantities of ROS through the Fenton reaction, which also causes ferroptosis [16]. These two processes are the main contributors to cell ferroptosis, however, other independent parallel pathways are also present.

### GPX4-GSH axis

The selenium oxidase GPX4 is the upstream limiting factor of ferroptosis. It converts GSH into L-Glutathione oxidized (GSSG) and reduces phospholipid hydroperoxides (PLOOHs) to their corresponding alcohols (PLOHS) [17]. The biosynthesis process of GPX4 is controlled by the mevalonate acid (MVA) pathway, and coenzyme Q10, synthesized via the MVA pathway, acts as an endogenous antioxidant and prevents ferroptosis in cells by decreasing lipid peroxidation [18]. GSH is a tripeptide consisting of glutamic acid, cysteine, and glycine. The glutamate-cysteine ligase catalytic (GCLC) is involved in the synthesis of GSH [19]. System-Xc is composed of solute carrier family 3 member 2(SLC3A2) and solute carrier family 7member 11(SLC7A11), which are embedded on the surface of the cell membrane. It facilitates the removal of glutamate from the cell and the transport of extracellular cystine into the cell for synthesizing GSH [12]. GSH is a reductant of GPX4 that interacts with GPX4 to protect cells from lipid peroxidation damage and ferroptosis.

### Iron metabolism

Iron overload causes cancer cells to undergo ferroptosis [20]. Transferrin (TF), transferrin receptor 1 (TFR1), ferroportin (FPN), divalent metal transporter 1 (DMT1), ferritin (ferritin heavy polypeptide 1 (FTH1), ferritin light chain (FTL)), and other iron metabolism-related proteins are all important carriers that participate in ferroptosis [21]. Ferric ions (Fe^3+^) are transported into cells by TFR1, and then, they are reduced to Fe^2+^ by the six transmembrane epithelial antigens of prostate 3(Steap3). DMT1 subsequently transports Fe^2+^ to form an unstable iron pool (LIP) to participate in iron death [20, 22].

Nuclear factor (erythroid-derived 2)-like 2 (Nrf2) is involved in the regulation of iron metabolism [21]. Besides upregulating FTH1, FPN, and heme oxygenase-1 (HO-1) to reduce the intracellular levels of ferrous ions, Nrf2 also raises increases the content of SLC7A11 and prevents ferroptosis [21]. Sequestosome 1 (SQSTM1, p62) enhances the inhibitory effect on ferroptosis by increasing the content of Nrf2 in the nucleus by inhibiting the degradation of Nrf2 by KELCH-like ECH-associated protein 1 (KEAP1) [23]. Autophagy-related protein 5 (ATG5) and ATG7 promote nuclear receptor coactivator 4 (NCOA4) to drive ferritin-selective autophagic degradation, raise iron levels and ROS production, and trigger ferroptosis [21, 24, 25]. Nitrogen fixation 1 homolog (S. cerevisiae) (NFS1) protein extracts sulfur from L-Cysteine to synthesize iron-sulfur clusters (ISCs). Inhibition of NFS1 triggers iron starvation, which accelerates the entry of ferrous ions into cells, and increases the sensitivity of these cells to ferroptosis [26, 27].

### Lipoid metabolism

Acyl-CoA synthetase long-chain family member 4 (ACSL4) and lysophosphatidylcholine acyltransferase 3 (LPCAT3) participate in the conversion of acyl-CoA (PUFA-CoA) into polyunsaturated fatty acid chains (PUFA-PLs). The suppression of ACSL4 can prevent breast cancer cells from ferroptosis [28, 29]. When PUFA-PLs are oxidized to lipid peroxides (PL-PUFA-OOH) under the action of arachidonate 15-Lipoxygenase (ALOX15), they also cause ferroptosis in cells [28]. Exogenous monounsaturated fatty acids (MUFAs), such as palmitoleic acid (POA), can block ferroptosis induced by erastin and RSL3 [30]. When MUFAs are activated by ACSL3, they replace PUFAs from phospholipids present in the plasma membrane and lessen the chances of the oxidation of lipids in the plasma membrane [31].

### Others

The GTP cyclohydrolase 1 (GCH1)-tetrahydrobiopterin (BH4) pathway is crucial in the regulation of ferroptosis, which is parallel to the GPX4 axis [31]. By utilizing reduced coenzyme II (Nicotinamide adenine dinucleotide phosphate, NADPH), ferroptosis suppressor protein 1 (FSP1) catalyzes the formation of ubiquinone from coenzyme Q10 [30]. The FSP1-CoQ10 axis restraints phospholipid peroxidation and shields cells from ferroptosis [32]. Ion and metabolite transport is facilitated by voltage-dependent anion channels (VDACS) in the mitochondria [33]. Erastin causes cells to undergo ferroptosis by modulating VDACS [33]. Mitochondrial respiration and its associated products also induce ferroptosis by lipid peroxidation [12]. Non-coding RNA (ncRNA) is crucial during tumor development [34]. Among them, microRNA (miRNA) and long non-coding RNA (lncRNA) might be targets for ferroptosis aiming to exert an anti-tumor effect [25, 35].

## Ferroptosis-associated genes in tumors

### p53

As a tumor suppressor, p53 is known as the “guardian of the genome”. It controls ferroptosis after transcription or translation [36]. The p53 protein positively controls ferroptosis by interacting with SLC7A11 to prevent the production of GSH, activate the expression of SAT1, and promote the activity of ALOX1 [32, 37, 38]. Additionally, when cystine is depleted in cancer cells, p53 controls the p21 protein (Cyclin-Dependent Kinase Inhibitor 1A, CDKN1A) to promote the accumulation of intracellular GSH and prevent ferroptosis [38, 39]. The p53 protein also inhibits lipid peroxidation by blocking the interaction of DPP4 with NADP [25, 40].

### RAS

Ferroptosis was first identified while searching for a small-molecule drug targeting the HRas Proto-Oncogene, GTPase (HRAS) ^G12V^ gene [41]. Different subtypes of RAS can control ferroptosis by inducing NADPH-oxidase (NOX) [41]. The KRAS proto-oncogene, GTPase (KRAS) ^G12V^, activates and upregulates NOX4 after the inactivation of the tumor suppressor cyclin-dependent kinase inhibitor 2A (p16). This increases intracellular ROS levels, which affects lipid peroxidation and ferroptosis [42]. KRAS^SG12D^ activates nuclear Nrf2 to promote the clearance of redox-active iron and plays a protective role during ferroptosis [43]. The overexpression of RAS mutations in rhabdomyosarcoma cells also increases resistance to ferroptosis induced by erastin and RLS3 [43].

### Other genes

**Fig. 1:**
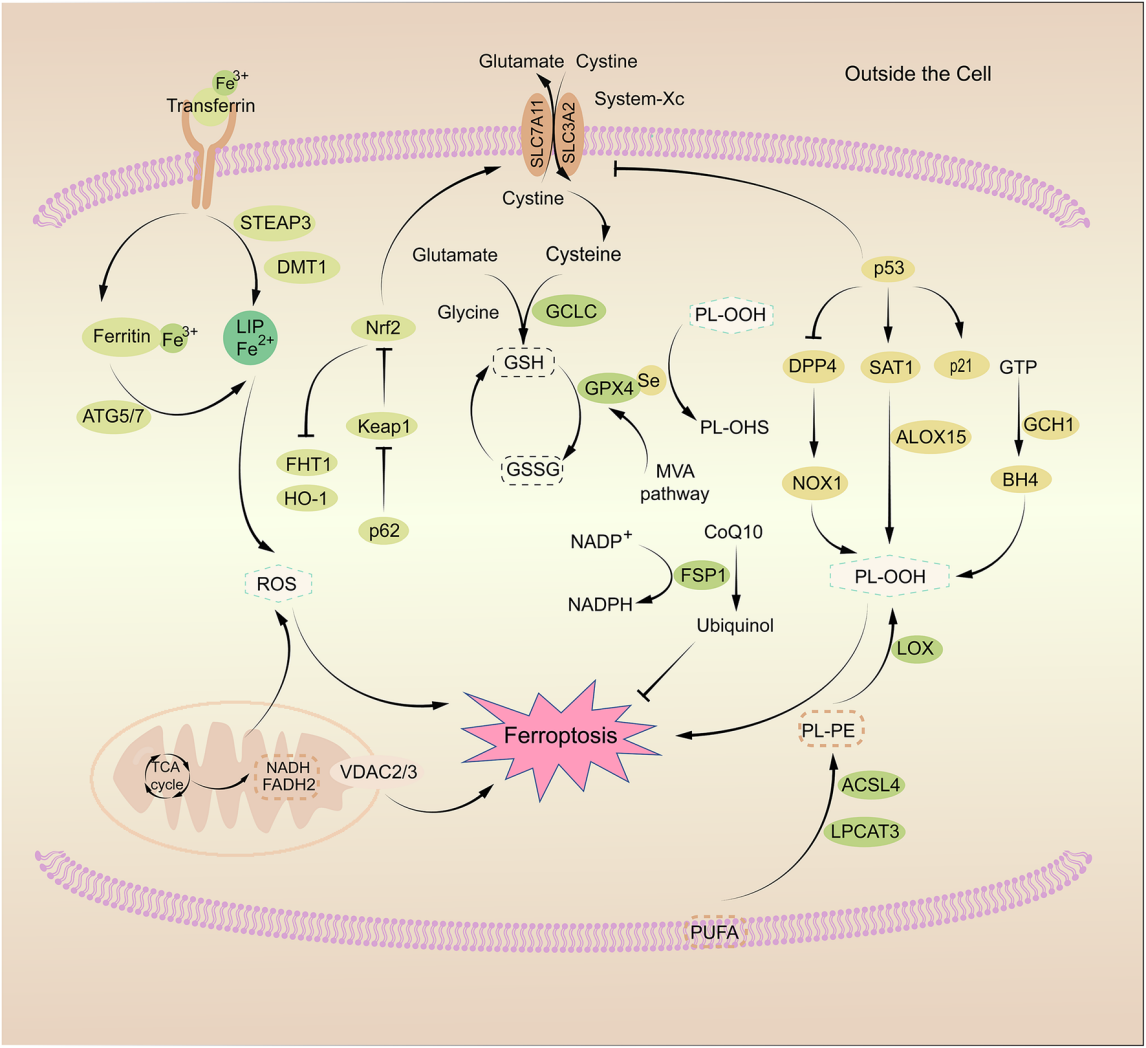
Schematic diagram of the mechanism of ferroptosis. Transferrin, transferrin receptor 1, and other iron metabolism-related proteins regulate ferroptosis by affecting the labile iron pool (LIP). p53 positively controls ferroptosis by inhibiting System-Xc and activating spermidine/spermine N1-acetyltransferase 1 (SAT1) and ALOX1, negatively regulating ferroptosis by controlling p21 and blocking dipeptidyl peptidase-4 (DPP4). System-Xc influences the synthesis of GSH, which interacts with GPX4 to protect cells from lipid peroxidation damage and ferroptosis. ACSL4 and LPCAT3 are involved in the synthesis of PUFA-CoA into PUFA-PLs, which can cause ferroptosis when oxidized to PL-PUFA-OOH. Mitochondrial respiration and its associated products also induce ferroptosis by lipid peroxidation

Autophagy can be induced by oxidative stress and the products of lipid peroxidation; excessive autophagy may lead to ferroptosis [44]. The target of rapamycin (mTOR) is a negative regulator of autophagy. It is positively associated with GPX4 levels and inhibits autophagy-dependent ferroptosis [45]. The gene of cAMP response element-binding protein (CREB) is highly expressed in tumor tissues and controls the production of GPX4 [46]. The Hippo pathway negatively modulates some transcription factors, including the Yes-associated protein 1 (YAP1) and the WW domain-containing transcription regulator 1 (TAZ) [47]. Both promote ferroptosis in human renal cell carcinoma or ovarian cancer cells by promoting iron accumulation and lipid peroxidation [47]. The mitogen-activated protein kinase (MAPK) family also plays an important role in erastin-induced ferroptosis in cancer cells [48] (Fig. 1).

## Targeting ferroptosis against cancer

The metabolic pathways in cancer cells undergo extensive reprogramming to meet their increased energy and biosynthetic demands and support their rapid proliferation [49]. This metabolic reprogramming often leads to unique metabolic features such as enrichment of PUFA-PLs (polyunsaturated fatty acid-containing phospholipids) and iron overload, which can create vulnerabilities in cancer cells that can be targeted for iron-dependent cell death [50]. Ovarian cancer cells have the feature of ferroptosis susceptibility since their tumor-initiating cells (TIC) overexpress TFR1 to overload intracellular iron [51]. Thus, ovarian cancer cells are predisposed to ferroptosis in response to medication [51]. Moreover, studies have also shown that inducing ferroptosis can reverse drug resistance, which is achieved by modulating the GPX4 pathway, iron metabolism pathway, and lipid metabolism pathway [52]. Additionally, researches have demonstrated that GPX4 dependence makes drug-resistant breast cancer cells vulnerable to ferroptosis brought on by GPX4 inhibition [53]. Patients with advanced gastric cancer are generally treated with chemotherapy drugs, including cisplatin, but the tumor cells tend to develop resistance to cisplatin [54]. There is evidence that ferroptosis is linked to chemotherapy resistance in gastric cancer. Ferroptosis is induced by the elevated level of activating transcription factor 3 (ATF3) inhibiting the Nrf2/KEAP1/SLC7A11 signaling pathway in gastric cancer cells, which alleviates cisplatin resistance [55].

### Ferroptosis-associated small molecule drugs against cancer

Sorafenib is used as the first-line drug for treating advanced liver cancer. It can induce ferroptosis by inhibiting System-Xc [56]. Altretamine can enhance ROS accumulation and induce cancer cell ferroptosis by inhibiting GPX4 activity [41, 57–59]. Statins can prevent the biosynthesis of GPX4 by blocking the MVA pathway, which inhibits cancer cells [25, 60, 61]. Sulfasalazine (SAS) can inhibit the System-Xc protein and cause ferroptosis in breast cancer cells and growth inhibition in non-Hodgkin lymphoma [62, 63]. Lapatinib and neratinib, which are used for breast cancer treatment, lead to ferroptosis in cancer cells by increasing intracellular iron levels [64, 65].

In a study, erastin induced ferroptosis in HT-1080 cells by not only inhibiting SLC7A11 to block the uptake of cystine [55], but also by inhibiting VDACS and altering the permeability of the outer mitochondrial membrane [33]. However, further studies are needed to determine its therapeutic capabilities. RSL3 can induce ferroptosis and lipid peroxidation by regulating GPX4 expression in head and neck cancer cells [66]. The inhibition of Nrf2 can increase the susceptibility of drug-resistant cells to RSL3 [67]. FIN56 can induce the degradation of the GPX4 protein via acetyl-CoA carboxylase or the activation of squalene synthase (SQS) to deplete CoQ10, which can lead to ferroptosis [8, 68] (Table1).

**Table 1:** Small-molecule drugs targeting ferroptosis against cancer

Name	Target	Is it approved by clinical	Literature
Sorafenib	System-Xc	Yes, treatment of advanced liver cancer	[56, 69]
Altretamin	GPX4	Yes, treatment of ovarian cancer	[41, 58, 70]
APAP	GPX4	Yes, analgesic and antipyretic drugs	[59]
TZD	ACSL4	Yes, hypoglycemic drugs	[71]
Statins	GPX4	Yes, lipid-lowering drugs	[25, 60, 61]
SAS	System-Xc GPX4	Yes, treatment of rheumatoid arthritis and inflammatory bowel disease	[41, 62, 63]
Lapatinib	Transferrin FPN	Yes, treatment of breast cancer	[64]
Neratinib	Transferrin FPN	Yes, the treatment of solid tumors and metastatic breast cancer	[65]
AFC	GPX4	Yes, treatment of iron deficiency anemia	[57]
RSL3	GPX4	No	[67]
FIN56	GPX4 CoQ10	No	[8, 68]
FINO2	Oxidize intracellular iron, indirectly inhibit GPX4	No	[72]
Erastin	System-Xc VDAC	No	[33, 41, 56]

## Traditional Chinese medicine targeting ferroptosis against cancer

Radiotherapy and chemotherapy damage normal tissues and cells to some extent. Also, the ability of chemotherapeutic agents to kill tumor cells is limited by the emergence of resistance to chemotherapy [73]. Therefore, the molecular mechanism of cancer needs to be elucidated and new therapeutic agents need to be developed. Ferroptosis might be a promising strategy for treating malignant tumors and reversing drug resistance. As some studies have found that certain TCM interfere with ferroptosis, searching for drugs based on TCM that can target ferroptosis against cancer is a new and promising direction of research on antineoplastic drugs [74].

### Prescriptions of traditional Chinese medicine

Shuganning injection (SGNI) contains extracts of *Ganoderma Lucidum* (Leyss. Ex Fr.) Karst, *Isatidis* Radix, *Gardeniae* Fructus, *Artemisiae Scopariae* Herba, and the flavone glycoside baicalin [75]. In a study, SGNI upregulated HO-1 and LIP, which in turn increased ROS levels and led to the ferroptosis of cancer cells. It also significantly attenuated the growth of MDA-MB-231 cell xenografts in nude mice [75]. Fuzhengkang'ai decoction (FZKA) was found to be effective in the treatment of non-small-cell lung carcinoma. FZKA contains *Ophiopogonis* Radix, *Codonopsis* Radix, and *Astragali* Radix [76]. FZKA was also found to decrease the level of GPX4 protein and mRNA and induce ferroptosis in cancer cells by increasing lipid peroxidation and intracellular levels of ferrous ions, which was also found in vivo [11]. Yiqi Huayu decoction (YQHY) contains *Astragali* Radix, *Salviae Miltiorrhizae* Radix et Rhizoma, *Curcumae* Rhizoma, and other substances used in traditional Chinese medicine [77]. Studies have found that YQHY can decrease the content of GSH in gastric cancer cells and induce ferroptosis by affecting the expression of ACSL4 and related proteins, such as p53 [77].

### Chinese herbs and extracted parts

*Scutellaria barbata* Herba is a traditional antipyretic and detoxifying Chinese medicine with antibacterial and anticancer properties [78]. It can decrease the level of the ferroptosis inhibitors GPX4 and SLC7A11 and increase the level of the ferroptosis inducer ACSL4 in hepatoma cells [79]. It can also regulate lipid peroxidation and iron metabolism to induce ferroptosis [79]. The root of *Actinidia chinensis* Planch (ACP) has anti-tumor and hemostatic properties [80]. ACP can downregulate the expression of GPX4 and SLC7A1, which can lead to ferroptosis and inhibit the growth of human hepatoma cells [81]. Additionally, the ethanol extract of *Camellia nitidissima* Chi (CNC) and *Lycium barbarum* polysaccharide (LBP) can also inhibit several tumor cells by decreasing the level of expression of GPX4 and SLC7A11 proteins to boost ROS accumulation [82, 83]. Tian et al. found that Huaier aqueous extract induced ferroptosis in NCI-H1299 cells by causing ROS accumulation, and deferoxamine and ferrostatin-1 decreased the sensitivity of cancer cells to Huaier aqueous extract [84].

### TCM monomers

#### Terpenoids

Artemisinin extracted from *Artemisia annua* L. is the most widely studied terpenoid compound that induces ferroptosis in TCM [85]. Artemisinin can selectively kill cancer cells and induce ferroptosis in RAS-mutant pancreatic cancer cells and leukemic cells [86, 87]. It causes ROS and iron-dependent cytotoxic effects on ovarian cancer cells and damage cancer cells through the co-administration of cell cycle blockers [88, 89]. Artemisinin induces ferroptosis in cells by stimulating ferritin degradation in the lysosome to produce free iron and influences the mitochondrial electron transport chain to stimulate ROS production from various tumor sources [90].

Ursolic acid activates autophagy to degrade ferritin, which can lead to ferroptosis in cancer cells via the induction of iron overload [91]. The combination of ursolic acid and cisplatin was found to significantly inhibit the growth of tumors and decrease adverse effects [91]. By blocking the PKR-like ER kinase (PERK)-Nrf2-HO-1 signaling pathway, tagitinin C can promote lipid peroxidation and ferroptosis in colon cancer cells [92]. Triptolide can also affect this pathway. It induces ferroptosis in head and neck cancer cells by downregulating the expression of Nrf2 and its target gene SLC7A11 [93]. Additionally, cucurbitacin B, glycyrrhetinic acid, and ophiopogonin B can increase lipid peroxidation levels and cause ferroptosis in cancer cells through GPX4-GSH-related pathways [94–96].

Other terpenoids can also induce ferroptosis in cancer cells through multiple pathways. For example, curcumenol decreased FTH1 levels by targeting miR-19b-3p via lncRNA H19 while inhibiting some factors, such as GPX4 and Nrf2, to increase ROS levels to trigger ferroptosis in lung cancer cells [10]. Oleanolic acid inhibits FTH1 and GPX4 proteins while increasing ACSL4 and TFR1 expression, causing Fe^2+^ and ROS accumulation to induce ferroptosis in HeLa cells [97]. β-element, along with cetuximab, can induce the depletion of GSH in KRAS mutant colon cancer cells, increase lipid peroxidation, and decrease iron metabolism-related proteins, which can lead to iron accumulation and ferroptosis [98].

#### Flavonoids

In a study, chrysin was found to degrade FTH1 by inhibiting the activity of carbonyl reductase 1(CBR1), which increased the levels of Fe^2+^ and lipid peroxidation and led to ferroptosis in pancreatic cancer cells [99]. Baicalin was also found to increase the iron levels in bladder cancer cells and cause ferroptosis by degrading FTH1 [100]. Ginkgetin and nobiletin can block the Nrf2/HO-1 signaling pathway. Ginkgetin was found to decrease the GPX4 protein levels and inhibit the antioxidant defense system of cells to cause ferroptosis in melanoma cells, whereas nobiletin reversed cisplatin resistance and increased lipid peroxidation and LIP levels in lung cancer cells [101, 102].

Quercetin can inhibit various cancer cells and induce ferroptosis by activating lysosomes to degrade ferritin, promote the release of iron, and enhance lipid oxidation [103, 104]. Molecular docking studies suggested that robuflavone A (RF-A), a novel Robusta biflavone compound obtained from *Selaginella trichoclada*, can bind to the E3 ubiquitin ligase NEDD4 to decrease its expression [105]. It can promote lipid peroxidation in mitochondria by inhibiting the degradation of VDAC2 and, thus, induce ferroptosis in cancer cells [106]. Additionally, auriculasin was found to cause mitochondrial shrinkage and ferroptosis in colon cancer cells by increasing intracellular ROS levels [107].

#### Phenolic compounds

Curcumin is the active component of *Curcuma longa* Rhizoma. It was found to prevent the proliferation of sunitinib-resistant clear cell renal cell carcinoma (CCRCC) cells by decreasing the levels of FTH1 and p53 mRNA and protein; this effect can be prevented by ferroptosis inhibitors [108]. In a study, 6-Gingerol stimulated autophagy in lung cancer cells and caused ferroptosis by increasing the content of ROS and Fe^2+^ in the cells [109]. Gallic acid was found to inhibit the growth of colon cancer cells [110]. It significantly decreased the expression of GPX4 and SCL7A11, while enhancing TFR1 levels and increasing intracellular Fe^2+^ and ROS, thus promoting ferroptosis [110].

#### Quinones

A group of quinones from *Salvia miltiorrhiza* Radix et Rhizoma, including dihydroisotanshinone I, tanshinone II, and cryptotanshinone, were found to stimulate ferroptosis in tumor cells. Dihydroisotanshinone I induced ferroptosis in two types of cancer cells by increasing intracellular lipid peroxidation and inhibiting the expression of the GPX4 protein [111]. It also significantly decreased the final tumor volume in two types of tumor-transplanted nude mice [112]. Tanshinone II upregulated the expression of p53 and decreased intracellular GSH and L-Cysteine levels. It stimulated ferroptosis in gastric cancer cells by increasing cellular lipid peroxidation and ROS levels [113]. Cryptotanshinone was found to induce ferroptosis in various cancer cell types by inhibiting the levels of SCL7A11, GPX4, and FPN and increasing the accumulation of ROS [114, 115].

#### Other compounds

By controlling the p53/SLC7A11/GPX4 signaling pathway, gambogic acid can disrupt cellular redox homeostasis and increase intracellular ROS and malondialdehyde (MDA) levels, thus inducing ferroptosis in different types of cancer cells [116, 117]. Bufotalin and matrine participate in this pathway. Bufotalin induces cancer cell ferroptosis by promoting GPX4 cellular degradation and increasing intracellular Fe^2+^ content. Matrine can considerably decrease the GSH content and the expression of GPX4 and SLC7A1 to inhibit the proliferation of tumor cells [118, 119].

Ruscogenin was found to considerably decrease the activity of pancreatic cancer cells by altering the levels of TFR1 and FPN, which promoted iron accumulation and induced ferroptosis in cancer cells [120]. Erianin can also facilitate an increase in intracellular iron levels and stimulate Ca^2+^ absorption by influencing the calcium-regulatory protein calmodulin (Cam) [121]. It was found to trigger ferroptosis in lung cancer cells by increasing ROS production and the level of Fe^2+^ [121]. Atractylodin was found to induce ferroptosis in hepatocellular carcinoma (HCC) cells by suppressing the expression of GPX4 and activating the ACSL4 and TFR1 proteins [122]. Piperlongumine can kill breast cancer cells by increasing intracellular ROS levels and then inducing ferroptosis [123].

### Monomer derivatives of TCM

**Fig. 2:**
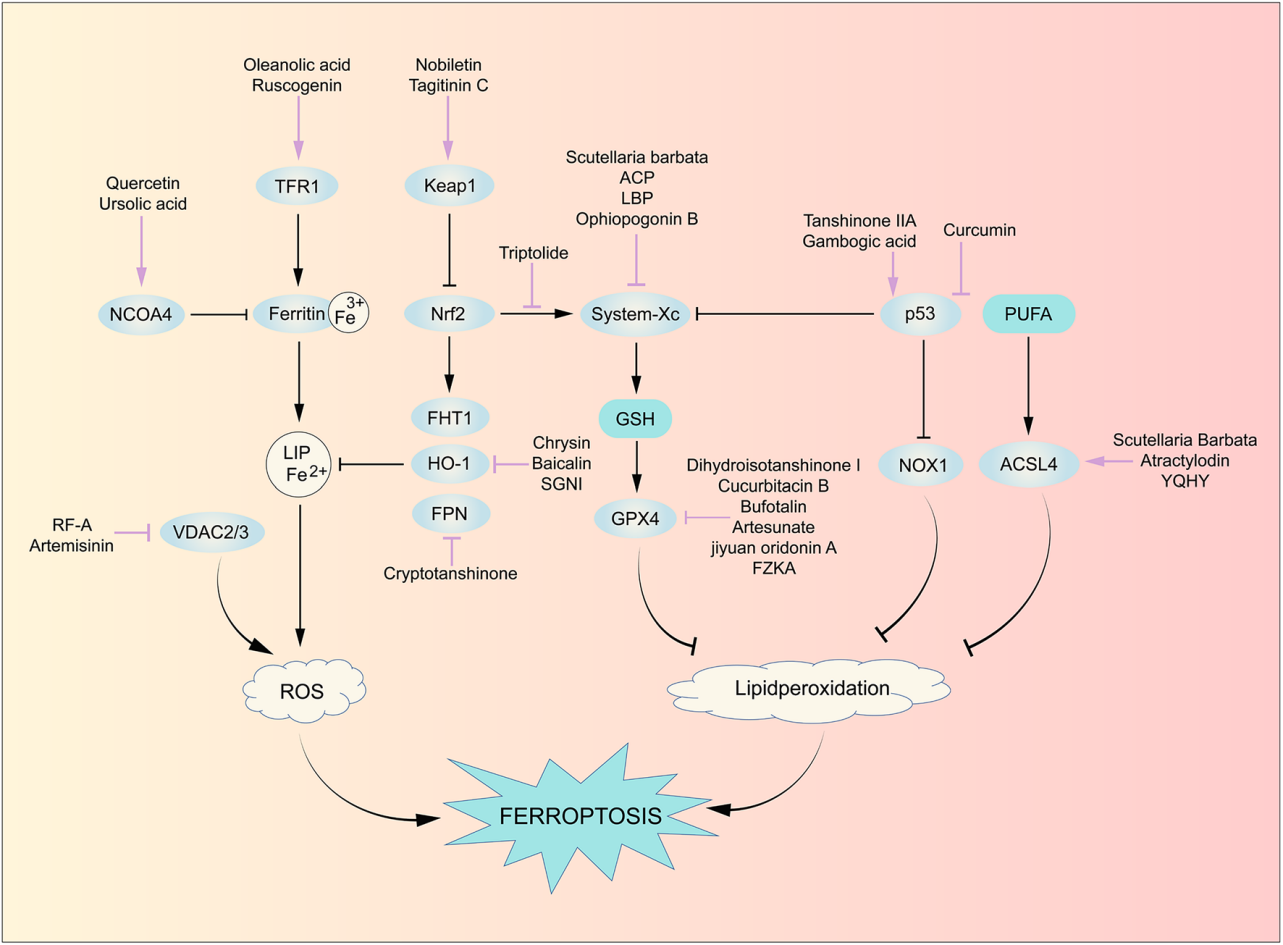
TCM targeting ferroptosis against cancer. *ACP* the root of *Actinidia chinensis* Planch, *LBP Lycium barbarum* polysaccharide, *FZKA* Fuzhengkang'ai decoction, *YQHY* Yiqi Huayu decoction. Oleanolic acid, ruscogenin and other compounds induce ferroptosis by affecting iron metabolism. Nobiletin, tagitinin C and other compounds act on the p62-Keap1-Nrf2pathway to lead to ferroptosis. *Scutellaria barbata*, dihydroisotanshinone I and other Chinese medicine result in ferroptosis by functioning in GPX4-GSH axis. Tanshinone IIA, gambogic acid and curcumin induce ferroptosis by modulating p53. Atractylodin and others cause lipidperoxidation by activating ACSL4

Dihydroartemisinin and artesunate are compounds derived from artemisinin, and they have biological properties similar to those of artemisinin [89]. Dihydroartemisinin and sorafenib work synergistically to increase the levels of ROS and decrease the levels of proteins, including GPX4, thus, inducing ferroptosis in hepatoma cells [124]. Artesunate can induce ferroptosis by increasing ROS production, lowering GPX4 expression, depleting intracellular GSH, and inducing iron deficiency to prevent the growth of sunitinib-resistant renal cancer cells [125]. Liu et al. found that A2, a derivative of jiyuan oridonin A, caused ferroptosis by decreasing the level of expression of the GPX4 protein and mRNA in gastric cancer cells [126]. It also inhibited cell growth through autophagy-dependent iron accumulation [126] (Table 2 and Fig. 2).

**Table 2:** TCM targeting ferroptosis against cancer

Classification	Medicine	Mechanisms	Concentration in vivo or in vitro	Literature
TCM prescriptions	Shuganning injection	Increased HO-1, LIP, and ROS	MDA-MB-231 (10.47 μg/ml); Xenograft (112.5 mg/kg)	[75]
TCM prescriptions	Fuzhengkang'ai decoction	Inhibited GPX4, increased the level of Fe^2+^	H1299 (0.75 mg/ml); Xenograft (31 g/kg)	[11, 76]
TCM prescriptions	Yiqi Huayu decoction	Influenced ACSL4 and p53	AGS (11.20 mg/ml)	[77]
TCM	*Scutellaria barbata*	Inhibited GPX4 and SLC7A11 Up-regulated ACSL4	SMMC-7721, HepG2, Huh7 (44.26、42.19、52.01 μg/ml); Xenograft (140 g/10 g)	[78, 79]
TCM	ACP	Down-regulated GPX4 and SLC7A11	HGC-27; Xenograft in zebrafish embryos (90, 180 mg/ml)	[80, 81]
TCM extracted parts	The ethanol extract of CNC	Decreased GPX4、SLC7A11, FTH1 Increased P53、ACSL4	HCT116 (92.37 μg/ml); Xenograft (1.2, 2.4, or 4.8 g/kg/)	[83]
TCM extracted parts	LBP	Decreased SLC7A11, GPX4	MCF-7, MDA-MB-231(> 4.0 mg/ml)	[82]
TCM extracted parts	Huaier aqueous extract	Increased the level of ROS	NCI-H1299	[84]
Terpenes	Artemisinin	Promoted degrading ferritin in the lysosome, produced free iron; Induced ROS	Hela (50 μM)	[87, 90]
Terpenes	Ursolic acid	Activated autophagic degradation of ferritin, induced ferrous ions overload	HOS, 143B (35 μM UA and 20 μM CIS)	[91]
Terpenes	Tagitinin C	Activated the PERK-Nrf2-HO-1 pathway to increase lipid peroxidation	HCT116	[92]
Terpenes	Triptolide	Inhibited Nrf2 and SLC7A11	HK1, FaDu	[93]
Terpenes	Cucurbitacin B	Decreased GPX4	CNE1 (0.016 μM); Xenograft (0.5,1 mg/kg)	[95]
Terpenes	Glycyrrhetinic acid	Activated NOX, inhibited SLC7A11, decrease GPX4	MDA-MB-231 (71.07 μM)	[94]
Terpenes	Ophiopogonin B	Inhibited GPX4 and SLC7A11	AGS, NCI-N87 (21.32 μM); Xenograft (50 mg/kg)	[96]
Terpenes	Curcumenol	By lncRNA H19 targeting miR-19b-3p to increase FTH1	H1299, H460; Xenograft (200 mg/kg)	[10]
Terpenes	Oleanolic acid	Induced ACSL4 and TFR1, inhibited FTH1 and GPX4	Hela; Xenograft (40,80 mg/kg)	[97]
Terpenes	β-Elemene	Induced the product of ROS, consumed GSH	HCT116 (125 μg/ml); Xenograft (50 mg/kg)	[98]
Flavonoids	Chrysin	Increased the level of ROS, degrading FTH1	PANC-1	[99]
Flavonoids	Baicalin	Decreased FTH1, increased ROS	5637; Xenograft (200 mg/kg)	[100]
Flavonoids	Nobiletin	Inhibited Keap/Nrf2/HO-1 pathway, down-regulated GPX4	SK-MEL-28 (53.63 μM)	[102]
Flavonoids	Ginkgetin	Increased transferrin, caused lipid peroxidation, and improved the level of LIP	A549	[101]
Flavonoids	Quercetin	Degrading ferritin	HepG2, Hep3B, HCT116 (25,50 μM)	[103, 104]
Flavonoids	Robustaflavone A	Decreased Nedd4, increased the expression of VDAC	MCF-7 (11.89 μM)	[105, 106]
Flavonoids	Auriculasin	Increased the level of ROS	HCT116, SW480 (5 μM)	[107]
Phenols	Curcumin	Decreased NCOA4, FTH1, and p53	A498, 786-O (sunitinib-resistant)	[108]
Phenols	6-Gingerol	Inhibited USP14, increased the level of ROS and Fe^2+^	A549; Xenograft (0.5 mg/kg)	[109]
Phenols	Gallic acid	Inhibited GPX4 and SCL7A11, increased TFR1	HCT-116, Caco-2	[110]
Quinones	Dihydroisotanshinone I	Inhibited GPX4	MCF-7 (5,10 μM), A549 (20 μM); Xenograft (30 mg/kg)	[111, 112]
Quinones	Tanshinone IIA	Up-regulated p53, decreased GSH and cysteine, increased ROS	BGC-823 (2.8 μM); Xenograft in NOD-SCID mice (50 mg/kg)	[113]
Quinones	Cryptotanshinone	Down-regulated GPX4 and FPN	A549 (20 μM)	[114, 115]
Polyprenylated xanthone	Gambogic acid	Regulated p53/SLC7A11/GPX4 pathway, disrupted redox homeostasis, increased ROS	A375, PCAP-1 (185 nM)	[116, 117]
Steroids	Bufotalin	Inhibited GPX4	A549 (4.21 μM); Xenograft (5,10 mg/kg)	[118]
Alkaloids	Matrine	Decreased GPX4, SLC7A1, increased TFR2 and the levels of ROS, Fe^2+^	HCT116 (6.1 mM); Xenograft (0.32 mmol/kg)	[119]
Steroids	Ruscogenin	Regulated TFR and FPN, caused the accumulation of iron	BxPC-3 (7.32 μM); Xenograft (5,10 mg/kg)	[120]
Dibenzyl compound	Erianin	Acting on calcium-regulatory protein calmodulin, up-regulated Fe^2+^	H460 (50 nM); Xenograft (100 mg/kg)	[121]
Polyacetylenes	Atractylodin	Inhibited GPX4, increased ACSL4 and TFR1	Huh7 (22.36 μM), Hccm (59.71 μM)	[122]
Alkaloids	Piperlongumine	Increased the levels of ROS	MIAPaCa-2, PANC-1 (14 μM)	[123]
Sesquiterpenes	Dihydroartemisinin	Decrased GCLC, GPX4 and HO-1	HepG2 (16.16 μM); Xenograft (40 mg/kg)	[124]
Sesquiterpenes	Artesunate	Induced the product of ROS, decreased GPX4, consumed GSH	KTCTL-26 (17.79 μM)	[125]
Terpenes	Compound a2	Decreased GPX4 protein and mRNA, induced the accumulation of Fe^2+^	MGC-803; Xenograft (5, 10, 20 mg/kg)	[126]

## Discussion

In this review, we discussed various processes associated with ferroptosis, including the GPX4-GSH axis, iron metabolism, and lipid metabolism. The application of TCM to target ferroptosis is a promising approach in the treatment of cancer. We summarized the mechanisms underlying TCM-targeted ferroptosis in anti-tumor effect. Our study might act as a reference for further research on anti-cancer drugs that target ferroptosis.

Some malignant tumor cells are easily affected by conventional ferroptosis-inducing medications [52, 54]. Ferroptosis inducers combined with chemotherapeutic drugs make some resistant cells more sensitive, which indicates that targeting ferroptosis is a promising strategy for the treatment of cancer [52, 54]. However, ferroptosis can be beneficial and harmful as it can suppress the growth of its tumor and accelerate its occurrence [127]. The tumor suppressor gene p53 regulates ferroptosis bi-directionally, suggesting that ferroptosis also contributes to carcinogenesis in non-beneficial ways [39, 40]. Additionally, organelles like the endoplasmic reticulum might be involved in ferroptosis [128]. Other types of cell death, such as autophagy, are also associated with ferroptosis [129]. Thus, a therapeutic approach needs to be investigated that might be able to inhibit cancer cells by controlling the co-occurrence of ferroptosis and other processes of cell death, such as autophagy, apoptosis, and cell cycle arrest.

The application of TCM is an effective strategy for treating cancer. Thus, the effects of TCM on ferroptosis may be further investigated for cancer treatment. Many researchers are currently investigating the targeting of ferroptosis by small-molecule drugs [8, 71], the discovery and development of such drugs should be further encouraged. We summarized the TCM prescriptions, Chinese herbs, extraction parts, monomers, and monomeric derivatives with anti-tumor effects associated with the induction of ferroptosis and found that most of them were monomers. This might be due to their chemical structures, which facilitate the comprehensive analysis of their mechanism of action. TCM prescriptions, Chinese herbs, and some extraction parts have already been applied in clinical practice, the research on whether their mechanisms are related to ferroptosis is still under investigation. While they always have complex compositions, which contribute to their multi-target effects and characteristics acting on multiple pathways, a bottleneck is encountered when attempting to expand the clinical applications of these medicines by thoroughly studying the components and ferroptosis mechanisms within them. TCM monomers have advantages in drug development due to their well-defined chemical structures, meanwhile, because they have relatively clear mechanisms, monomers are easier to develop into targeted drugs. Additionally, it’s easier to study the pharmacokinetics of them. And monomers could have better efficacy and lower toxicity through structure optimization and drug design. Therefore, they have greater potential in drug development and clinical application. Studies on monomers obtained from traditional Chinese medicine are extremely important for developing novel medication, which requires the alteration of the structures of monomers to produce new molecules with improved bioavailability or lower IC_50_. Some small-molecule drugs are modified in nano-form to enhance the efficacy of the drugs or address other limitations; nanomaterials that target ferroptosis have advanced progress [130, 131]. By summarizing the different kinds of anti-cancer TCM that can induce ferroptosis, we aim to help researchers in this field identify anti-tumor active monomers derived from traditional Chinese medicine for developing prodrugs and encourage them to investigate new anti-tumor mechanisms. We found that some TCM can enhance ROS production and disrupt the redox balance in cancer cells [132]. Although the mechanism of action of certain drugs that target ferroptosis remains unknown, these drugs are promising therapeutic agents and should be further investigated.

Apart from monomers, TCM usually consists of multiple components, which can act through different pathways and targets. For example, the ethanolic extract of CNC can induce ferroptosis through various targets and pathways, such as GPX4 and SLC7A11 [81]. Small molecule compounds often have relatively single mechanisms of action, TCM may be more suitable for treating various types of tumors, such as those with low expression of specific target genes. The TCM monomers have unique characteristics and structures that are more distinct than small molecules. Some of these structures are difficult to synthesize chemically, but they can be obtained through extraction and isolation. Although TCM exerts its effects through multiple pathways and targets, it is important for us to continue seeking new mechanisms and not abandon in-depth research simply because of the identified existing mechanisms.

## Data Availability

Data sharing is not applicable to this article as no new data were created or analyzed in this study.

## Abbreviations

GPX4: Glutathione peroxidase 4
GSH: Glutathione
GCH1: GTP cyclohydrolase 1
BH4: Tetrahydrobiopterin
FSP1: Ferroptosis suppressor protein 1
TCM: Traditional Chinese medicine
SLC7A11: Solute carrier family 7 member 11
Nrf2: Nuclear factor (erythroid-derived 2)-like 2
ACSL4: Acyl-CoA synthetase long-chain family member 4
TF: Transferrin
TFR1: Transferrin receptor 1
ROS: Reactive oxygen species
PUFAs: Polyunsaturated fatty acids
LOXs: Lipoxygenases
GSSG: GSH into L-Glutathione oxidized
PLOOHs: Phospholipid hydroperoxide
PLOHS: Phospholipid hydroperoxide corresponding alcohols
MVA: Mevalonate acid
GCLC: Glutamate cysteine ligase catalytic
SLC3A2: Solute carrier family 3 member 2
SLC7A11: Solute carrier family 7member 11
FPN: Ferroportin
DMT1: Divalent metal transporter 1
FTH1: Ferritin heavy polypeptide 1
FTL: Ferritin light chain
Steap3: Six transmembrane epithelial antigens of prostate 3
LIP: Labile iron pool
HO-1: Heme oxygenase-1
p62: Sequestosome 1
KEAP1: KELCH-like ECH-associated protein 1
ATG5: Autophagy related proteins 5
ATG7: Autophagy related proteins 7
NCOA4: Nuclear receptor coactivator 4
NFS1: Nitrogen fixation 1 homolog (S. cerevisiae)
ISCs: Iron-sulfur clusters
LPCAT3: Lysophosphatidylcholine acyltransferase 3
PUFA-CoA: Acyl-CoA
PUFA-PLs: Polyunsaturated fatty acid chains
PL-PUFA-OOH: Lipid peroxides
ALOX15: Arachidonate 15-Lipoxygenase
MUFAs: Exogenous monounsaturated fatty acids
POA: Palmitoleic acid
VDACS: Voltage-dependent anion channels
ncRNA: Non-coding RNA
miRNA: MicroRNA
lncRNA: Long non-coding RNA
p21: Cyclin dependent kinase inhibitor 1A
HRAS: HRas Proto-Oncogene, GTPase
NOX: NADPH-oxidase
KRAS: KRAS proto-oncogene, GTPase
mTOR: Mechanistic target of rapamycin
CREB: CAMP response element-binding protein
YAP1: Yes-associated protein 1
TAZ: Transcription regulator 1
MAPK: Mitogen activated kinase-like protein
SAT1: Spermidine/spermine N1-acetyltransferase 1
DPP4: Dipeptidyl peptidase-4
TIC: Tumor-initiating cells
ATF3: Activating transcription factor 3
SAS: Sulfasalazine
SQS: Squalene synthase
PSAF NCS: Polyethylene glycol iron atom nanocatalysts
FZKA: Fuzhengkang'ai decoction
YQHY: Yiqi Huayu decoction
ACP: *Actinidia chinensis* Planch
CNC: *Camellia nitidissima* Chi
LBP: *Lycium barbarum* polysaccharide
PERK: PKR-like ER kinase
CBR1: Activity of carbonyl reductase 1
CCRCC: Clear cell renal cell carcinoma
MDA: Malondialdehyde
Cam: Calmodulin
HCC: Hepatocellular carcinoma
